# The Apgar Score and Infant Mortality

**DOI:** 10.1371/journal.pone.0069072

**Published:** 2013-07-29

**Authors:** Fei Li, Ting Wu, Xiaoping Lei, Hao Zhang, Meng Mao, Jun Zhang

**Affiliations:** 1 Ministry of Education-Shanghai Key Laboratory of Children’s Environmental Health, Xinhua Hospital, Shanghai Jiao Tong University School of Medicine, Shanghai, China; 2 Shanghai Institute of Pediatric Translational Medicine, Shanghai Children’s Medical Centre, Shanghai Jiao Tong University School of Medicine, Shanghai, China; 3 Chengdu Women's and Children's Central Hospital, Chengdu, Sichuan Province, China; College of Pharmacy, University of Florida, United States of America

## Abstract

**Objective:**

To evaluate if the Apgar score remains pertinent in contemporary practice after more than 50 years of wide use, and to assess the value of the Apgar score in predicting infant survival, expanding from the neonatal to the post-neonatal period.

**Methods:**

The U.S. linked live birth and infant death dataset was used, which included 25,168,052 singleton births and 768,305 twin births. The outcome of interest was infant death within 1 year after birth. Cox proportional hazard-model was used to estimate risk ratio of infant mortality with different Apgar scores.

**Results:**

Among births with a very low Apgar score at five minutes (1–3), the neonatal and post-neonatal mortality rates remained high until term (≥ 37 weeks). On the other hand, among births with a high Apgar score (≥7), neonatal and post-neonatal mortality rate decreased progressively with gestational age. Non-Hispanic White had a consistently higher neonatal mortality than non-Hispanic Black in both preterm and term births. However, for post-neonatal mortality, Black had significantly higher rate than White. The pattern of changes in neonatal and post-neonatal mortality by Apgar score in twin births is essentially the same as that in singleton births.

**Conclusions:**

The Apgar score system has continuing value for predicting neonatal and post-neonatal adverse outcomes in term as well as preterm infants, and is applicable to twins and in various race/ethnic groups.

## Introduction

In 1952, Virginia Apgar proposed a score system as a rapid means of evaluating the clinical status of the newborn and the need for prompt intervention to establish breathing [1]. It is a simple evaluation system including five easily identifiable components–heart rate, respiratory effort, muscle tone, reflex irritability and color. Score of 0, 1, or 2 is assigned to each component, and the sum of scores of the five components is the total score. A total score of 7 or higher suggests that the condition of baby is good to excellent. The Apgar score system offers a standardized, effective, and convenient assessment for newborn infants. It has gained widespread application by obstetricians all over the world for more than half a century.

In recent years, doubts have been cast on the value of the Apgar score. Studies found that the Apgar score failed to predict specific neurologic outcomes of the term infants, a use for which it was never intended [2]. What’s more, it was once inappropriately adopted alone to diagnose asphyxia [3]. In order to place the Apgar score in its proper perspective, the Neonatal Resuscitation Program guidelines state that “Apgar scores should not be used to dictate appropriate resuscitation actions, nor should interventions for depressed infants be delayed until the 1 minute assessment.” [4] Furthermore, the Apgar score also has its own limitations. A number of factors may influence an Apgar score such as drugs, trauma, congenital anomalies, infections, hypoxia, hypovolemia, and preterm birth. Up to date, there are few consistent data on the significance of the Apgar score in preterm infants. Because elements of the score such as tone, color and reflex irritability partially depend on the physiologic maturity of the infants, this situation may lead to a healthy preterm infant with no evidence of asphyxia receiving a lower score only because of immaturity [3].

In this study, we evaluated if the Apgar score remains pertinent after more than 50 years of wide use and with wide availability of prompt neonatal care. We also assessed the value of the Apgar score in predicting infant survival, expanding from neonatal to post-neonatal period.

## Methods

The U.S. linked live birth and infant death datasets published by the National Center for Health Statistics (NCHS) and the Centers for Disease Control and Prevention (CDC), contain information from matching birth and death certificates for all infants born in the United States who died during their first year of life. These files provide demographic and health data for births occurring during the calendar year based on information abstracted from birth and fetal death certificates filed in vital statistics offices of the 50 states and the District of Columbia, Puerto Rico, the Virgin Islands, and Guam. Available information in these files included demographic characteristics of mothers, obstetric history, major pregnancy complications, maternal smoking, status of prenatal care, labor and delivery complications, and birth outcomes. Each state also provided to NCHS matching birth and death certificate numbers for each infant under 1 year of age who died in the state. NCHS used the matching numbers to extract final edited data from the NCHS natality and mortality statistical files. These data were linked to form a single statistical record, thereby establishing a national linked record file. After the initial linkage, NCHS returned lists of unlinked infant death records and records with inconsistent data between the birth and death certificates to each state. State additions and corrections were incorporated, and a final national linked file was produced [5]. Cause-of-death statistics in the linked live birth and infant death datasets are classified in accordance with the Manual of the International Statistical Classification of Diseases, Injuries, and Causes of Death, Ninth Revision (ICD–9) from 1995–1998 [6]. Later issues of the datasets included causes of death classified according to the ICD–10 [7]. More information about the data can be found at http://www.cdc.gov/nchs/linked.htm. We used data from 1995 to 2004. Since these files are anonymized public data, our Institutional Review Board does not require a review.

Smoking during pregnancy was not reported in California, Indiana, South Dakota, and New York State (except New York City) during the study period. These subjects were coded as missing on smoking. The variable of smoking was recoded as “nonsmokers” (0 cigarette per day), “light smokers” (1 to 10 cigarettes per day), and “heavy smokers” (more than 10 cigarettes per day), respectively. Education levels were divided into: <12 years (Less than high school), 12 years (High school), 12–16 years (college), ≥17 years (graduate school). Marriage status was classified as married and unmarried. Variable of “when the prenatal care started” was divided into groups of 1st trimester (1st–3rd month), 2nd trimester (4th–6th month), 3rd trimester (7th–9th month) and no prenatal care.

Two types of estimation of gestational age were recorded on the certificates: gestational age based on self-reported last menstrual period (LMP) and clinical estimate (CE). Deficiencies of LMP-based estimate are well established [8], [9]. Several methods for editing the LMP-based gestational age have been proposed to reduce misclassification. Recently, Qin et al. used a simple method in which the CE of gestational age is substituted for LMP-based gestational age when the difference between the two estimates is greater than two weeks (LMP/CE method) [10]. This method appears to be effective in correcting large errors in gestational age estimates. It has a further benefit that records are reclassified, rather than excluded altogether. Thus, in our study the gestational age assigned to each infant was based on the LMP/CE method. Preterm infants were defined as those born between 24–36 weeks of gestation, and term infants as those born at or after 37 weeks of gestation.

**Figure 1:**
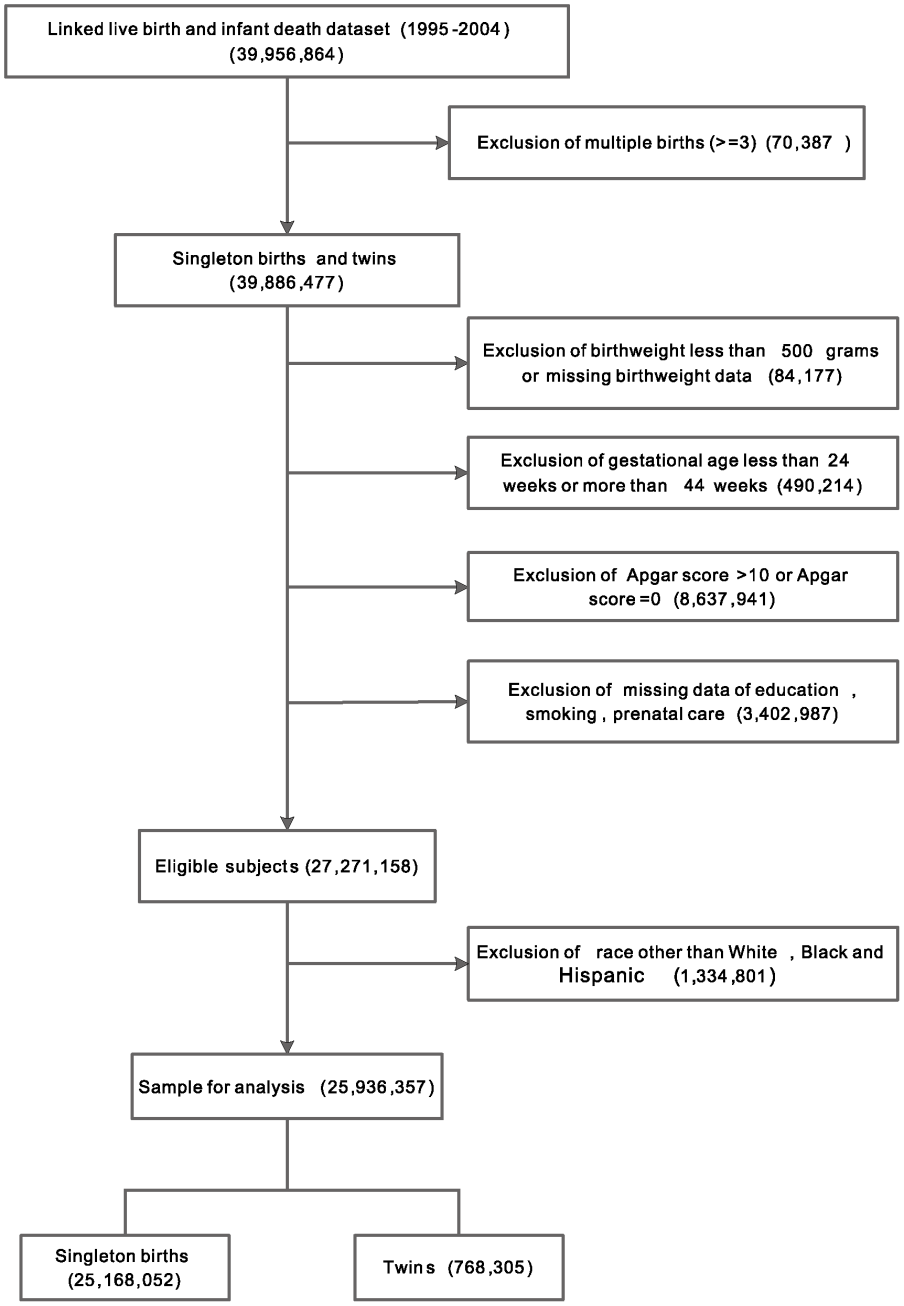
Flow chart of subjects included.

There were 39,956,864 live births in the linked 1995–2004 live birth and infant death dataset (Figure 1). Records were excluded in the following situations: triplets or higher order (70,387), births with less than 500 grams or with unknown birthweight (84,177), births at less than 24 weeks or longer than 44 weeks of gestation (490,214), and 5-minute Apgar score being 0, greater than 10 or missing (8,637,941). Records containing missing values of maternal education, time when prenatal care started, and maternal smoking (in states where smoking was recorded) were also excluded, leaving 27,271,158 births eligible for analysis. The number of races other than White, Black and Hispanic was too small, and therefore we also excluded them from analysis. The final sample size was 25,936,357, including 25,168,052 singleton births and 768,305 twin births.

The outcome of interest was infant death within 1 year after birth. All analyses were performed with Statistical Analysis System (version 9.2, SAS Institute, Cary, N.C.). Chi-Square test was used to compare infant mortality with different Apgar scores. Kaplan-Meier curve was performed to plot time to survival. Cox proportional hazard-model was used to estimate risk ratio of infant mortality comparing different Apgar scores, adjusting for potential confounders such as maternal education level, marital status, time when prenatal care started and maternal smoking during pregnancy. Non-Hispanic White was used as the reference group.

## Results

Table 1 shows the maternal characteristics of the study population. The mean maternal age was 27.3 years. The incidence of preterm birth (<37 weeks) was 9.8%. Two-thirds of women were non-Hispanic White. The vast majority started prenatal care in the first trimester. Table 2 presents the distribution of Apgar score among preterm, term and post-term births and neonatal and post-neonatal mortality rates. Preterm births had about 10 to 20-time higher incidence of low Apgar score at 5 minutes (<7) than term and post-term births, though the vast majority of preterm births had a score greater than 7. Both neonatal and post-neonatal mortality rates decreased with increasing Apgar score.

**Table 1:** Maternal characteristics of the study population.

	n	Proportion (%)
**Maternal age (mean±SD, y) Gestation week**	27.2±6.1	
24–25	49,353	0.18
26–27	66,965	0.25
28–29	91,405	0.34
30–31	152,833	0.56
32–33	307,781	1.13
34–36	2,012,283	7.38
37–41	23,712,439	86.95
41–44	878,099	3.22
**Maternal race**		
Non-Hispanic White	18,095,334	66.35
Non-Hispanic Black	4,540,838	16.65
Hispanic	3,300,185	12.10
other	1,334,801	4.89
**Maternal education level**		
<12 years(Less than high school)	5,128,628	18.81
12 years (High school)	8,841,014	32.42
13–16 years (college)	10,706,125	39.26
≥17 years (graduate school)	2,595,391	9.52
**Marital status**		
married	18,267,919	66.99
unmarried	9,003,239	33.01
**Time when prenatal care started (Month)**		
1st Trimester (1st–3rd month)	22,801,658	83.61
2nd Trimester (4th–6th month)	3,506,381	12.86
3rd Trimester (7th–9th month)	708,801	2.60
No prenatal care	254,318	0.93
**Maternal smoking during pregnancy**		
nonsmokers (0 cigarette per day)	24,019,159	88.08
light smokers (1 to 10 cigarettesper day)	2,294,467	8.41
heavy smokers (more than 10 cigarettesper day)	957,532	3.51

**Table 2:** Distribution of Apgar score at 5 minutes in preterm, term & post-term births and corresponding neonatal & post-neonatal mortality rates.

ApgarScore	Numberof Birth	Distribution of Apgar Score in			Neonatal MortalityRate (/1,000)	Post-neonatal MortalityRate (/1,000)
		Preterm Birth(24–36 weeks) (%)	Term Birth(37–41 weeks) (%)	Post-term Birth(42–44 weeks) (%)		
1	13,737	0.43	0.02	0.03	581.86	26.64
2	12,104	0.293	0.03	0.04	334.68	34.37
3	18,532	0.38	0.05	0.07	192.42	35.51
4	29,320	0.57	0.07	0.12	121.28	30.56
5	55,968	1.00	0.15	0.21	74.58	23.48
6	129,788	2.31	0.35	0.48	43.47	19.72
7	341,768	5.20	1.01	1.28	18.91	12.11
8	1,680,329	16.00	5.84	6.62	4.39	4.91
9	20,760,364	69.60	84.29	82.76	0.59	1.82
10	1,968,967	4.23	8.20	8.40	0.37	1.59

### Apgar Score and Neonatal and Post-neonatal Mortality

**Figure 2:**
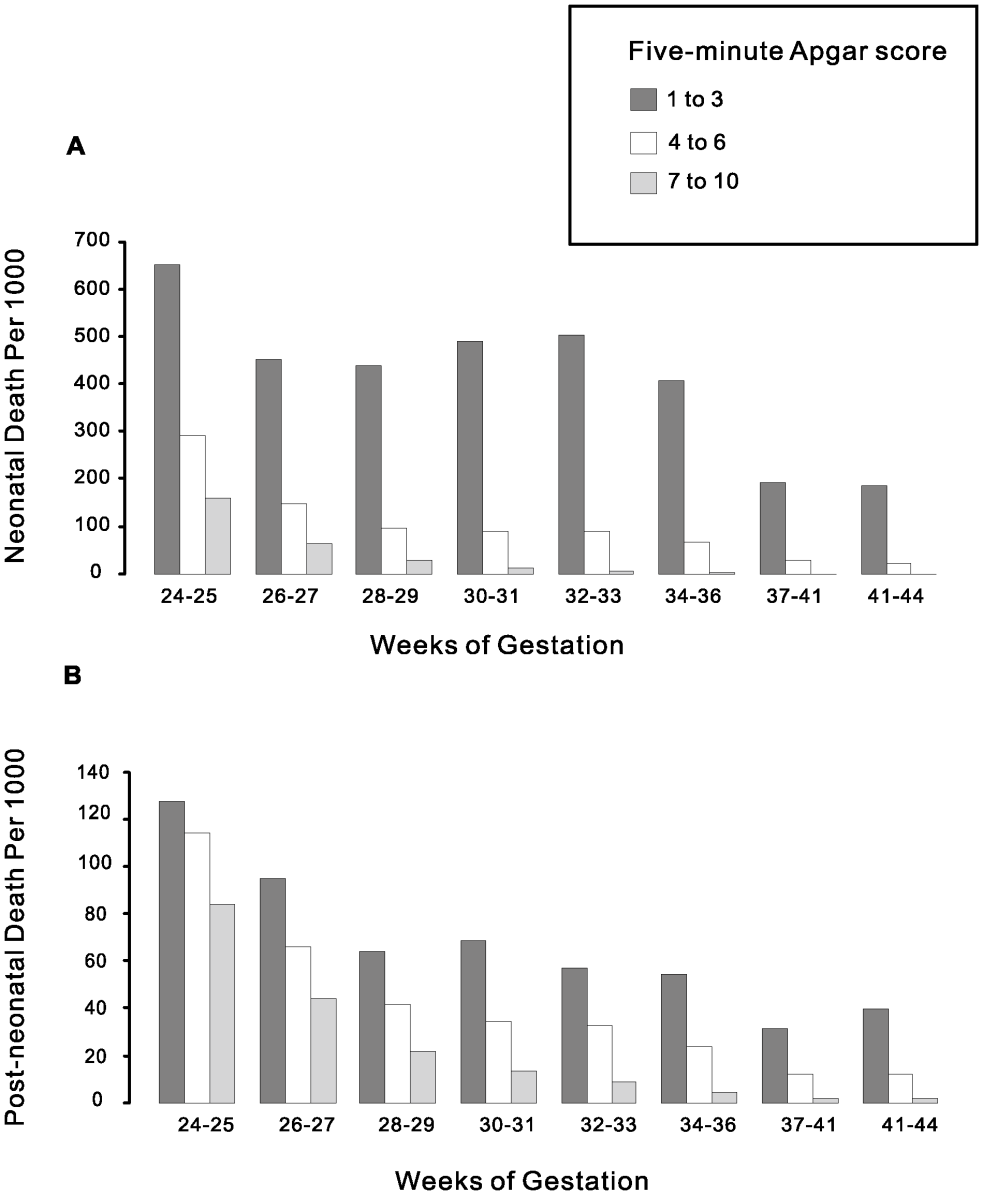
Neonatal (A) and post-neonatal mortality (B) by five-minute Apgar score and gestational age.

Figure 2 presents the neonatal and post-neonatal mortality by Apgar score and gestational age. Among births with a very low Apgar score (1–3), the neonatal mortality rate remained high until term (≥37 weeks). On the other hand, among births with a high Apgar score (≥7), neonatal mortality rate decreased progressively with gestational age (Figure 2A). This pattern was also observed for post-neonatal mortality (Figure 2B), indicating that low Apgar score is not closely related to immaturity. The differences in mortality rate by Apgar scores were all statistically significant.

### Apgar Score and Infant Mortality in Different Race/ethnic Groups

**Figure 3:**
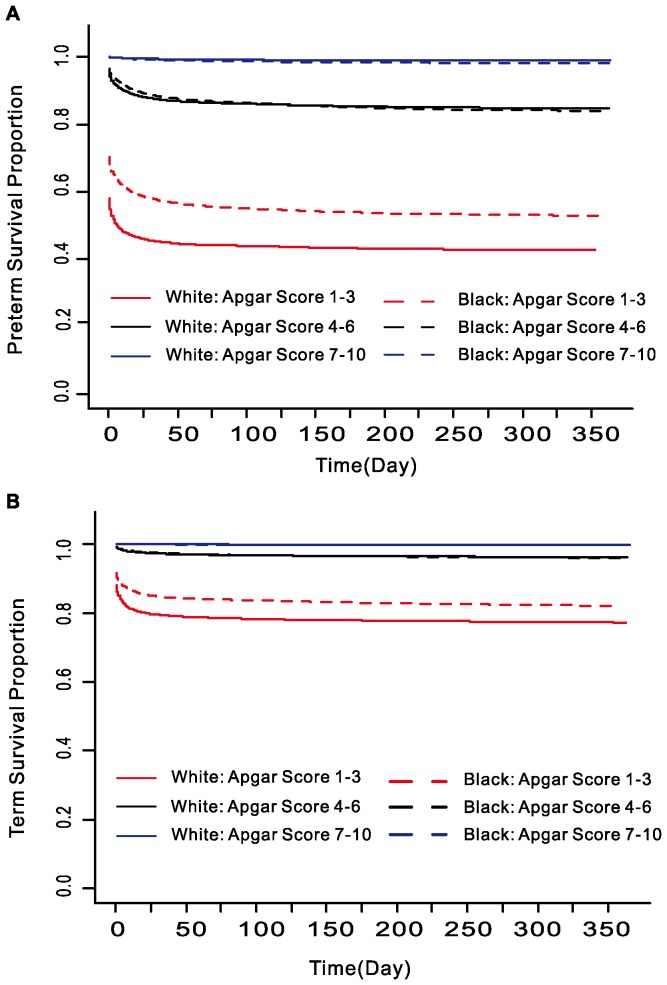
Survival curves in preterm (A) and term (B) births for White and Black infants by five-minute Apgar score from birth to 1 year.

Figure 3 depicts the survival curve among different Apgar scores in non-Hispanic White and Black separated by preterm (3A) and term (3B) births. For both preterm and term infants, the general trend of relationship was similar. At low Apgar score (1–3), Black had a significantly higher survival rate than White from birth to 1 year. But at higher Apgar scores, Black had a higher survival rate than White in neonatal period but a lower rate in post-neonatal period. This “cross-over” phenomenon was more obvious when we present it in a different way.

**Figure 4:**
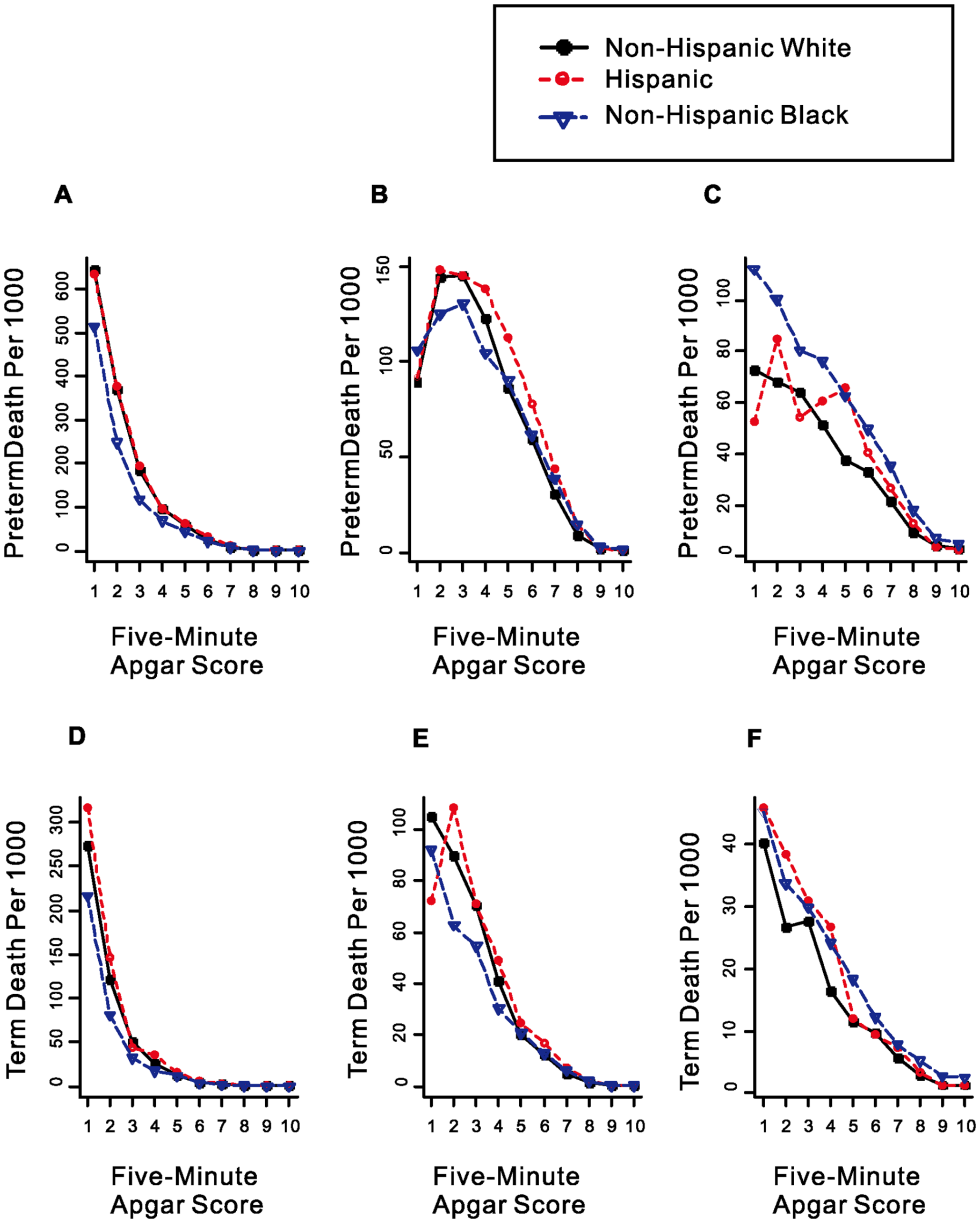
Mortality rates for White, Black and Hispanic infants by five-minute Apgar score at gestational age of 24–36 weeks (A–C) and 37–41 weeks (D–F). (A and D: Time of Infant Death≤1 Day, B and E: Time of Infant Death between 2 and 27 Days, C and F: Time of Infant Death≥28 Days).

Figure 4 illustrates how Apgar score performed in non-Hispanic White, non-Hispanic Black and Hispanics separated by preterm (A, B, C) vs term (D, E, F) and by death within 1 day (A, D), 2 to 27 days (B, E) and after 28 days (C, F) postnatal. For both preterm and term births, mortality rate within 1 day dropped precipitously with increasing Apgar score. After Apgar score reached 4 or above, further decrease in mortality rate slowed significantly. This was not the case in neonatal and post-neonatal mortality. The decrease did not slow until the Apgar score reached 7 or higher, suggesting that Apgar score is still a good predictor for neonatal (after 1 day) and post-neonatal death. Furthermore, non-Hispanic White had a consistently higher neonatal mortality than non-Hispanic Black in both preterm and term births. However, for post-neonatal mortality, Black had significantly higher rate than White.

To further explore the reason for the cross-over phenomenon, we compared mortality rate between Black and White, adjusting for maternal education, marital status, time when prenatal care started, and maternal smoking during pregnancy. The results confirmed what was observed in Figure 4 (Figure S1).

### Apgar Score and Twins/singleton Births Mortality

**Figure 5:**
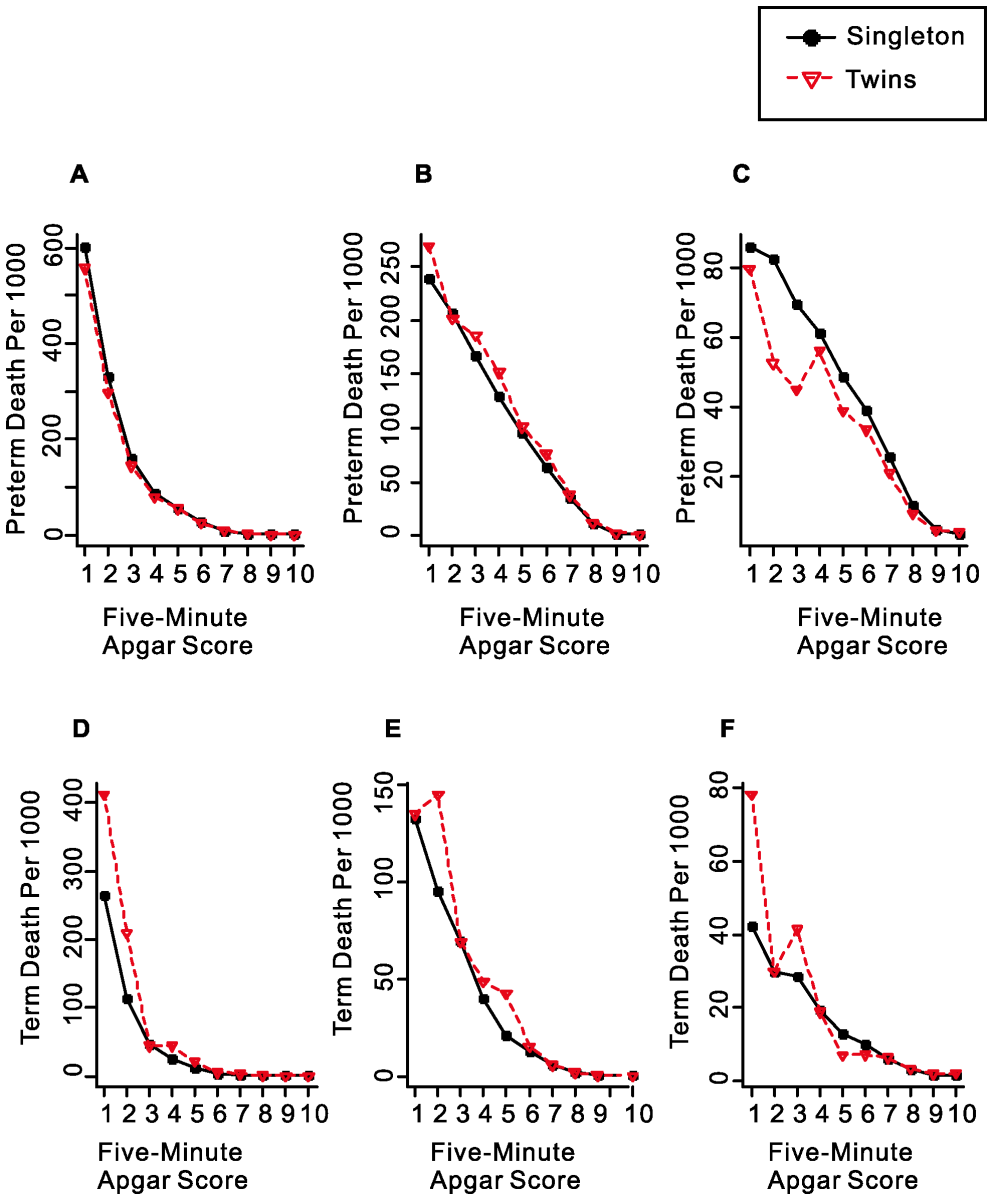
Mortality rates for singleton births and twins by five-minute Apgar score at gestational age of 24–36 weeks (A–C) and 37–41 weeks (D–F). (A and D: Time of Infant Death≤1 Day, B and E: Time of Infant Death between 2 and 27 Days, C and F: Time of Infant Death≥28 Days).

Finally, we examined whether Apgar score is as useful in twins as in singleton births. Figure 5 shows that the pattern of change in neonatal and post-neonatal mortality by Apgar score is essentially the same as that in singleton births, indicating that the Apgar score system is equally valid when it is applied to twins.

## Discussion

The Apgar score system was used to estimate the probability of survival of the infant [11], [12] and to appraise the need for resuscitation [1]. An additional score obtained at five minutes of age gained universal acceptance after the report from the Collaborative Perinatal Project showed a stronger relation between the five-minute score and neonatal mortality than the one-minute score [13]. However, it has been suggested that the Apgar score is antiquated and that its predictive value has been considerably weakened by the institution of prompt and effective neonatal care. Is the Apgar score still useful for the immediate assessment of neonates in contemporary practice?

Our analysis of the relationship between five-minute Apgar scores and infant survival indicates that the Apgar score is not only useful for neonatal period and term infants as it was 50 years ago, but also meaningful for post-neonatal period and preterm infants. We found that the Apgar score showed its predictive value for infant death of both very preterm, preterm and term infants in post-neonatal period. In fact, this long-term predictive value was similarly found in twins. Hence, Apgar score could still be a good and convenient predictor of infant death.

It is worth noting that the value of Apgar score, in predicting the infant death in either the neonatal or post-neonatal period, was influenced by race/ethnicity. At the same level of Apgar score, the mortality of Black newborns was substantially lower than White newborns in neonatal period, while the mortality of Black infants was consistently higher in post-neonatal period. This observed “cross-over” phenomenon in the unadjusted analysis was confirmed in both preterm and term gestation after adjusting for socioeconomic status (SES), approximated by maternal education, marital status and time when prenatal care started. Our findings are consistent with previous literature in that there is a substantial health disparity between races probably due to SES. On one hand, Black neonatal infants with lower SES may ironically have the advantage of fetal organ maturity over White neonatal infants especially in preterm period. It was hypothesized that corticotropin-releasing hormone (CRH) level may be higher in Blacks due to chronic stress or distress during pregnancy [14]. CRH triggers the release of fetal cortisol from the adrenals, which is a crucial stimulus of organ development [15]–[17]. Consequently, some organs such as fetal lung, were promoted to become mature sooner. On the other hand, Black post-neonatal infants with lower SES may be at a higher risk of low SES-related morbidity and mortality than White postnatal infants, such as infection, respiratory illness, impaired growth, inappropriate nutrition and poor social environment [18], [19]. While we tried to control for differences in SES, residual confounding may still exist because our SES measure may not include all measured and unmeasured variables that constitute a complex matrix of SES.

### Strengths and Limitations

Our study was based on a very large sample size, which allowed us to use neonatal and post-neonatal mortality as the outcomes. We were also able to validate the Apgar score system in various race/ethnic groups, in twin pregnancies and preterm births. However, the study also has some limitations. If an infant was born in a very severe condition and died quickly after birth, he/she may have been reported as a stillbirth. This situation may lead to an artificially lower neonatal mortality rate than it should be.

In summary, our findings support the continuing value of assigning Apgar score to predict neonatal and post-neonatal adverse outcomes in term as well as preterm infants. The Apgar score system is applicable to twins and in various race/ethnic groups.

## Supporting Information

Figure S1 **Relative risks of infant death for non-Hispanic Black vs non-Hispanic White (Reference) by Apgar score at five minutes after adjusting for maternal education, marital status, time when prenatal care started, and maternal smoking during pregnancy.**
(EPS)
